# *N*′-(Butan-2-yl­idene)furan-2-carbohydrazide

**DOI:** 10.1107/S1600536810038018

**Published:** 2010-09-30

**Authors:** Bu-wei Ma, Zhen-xin Zhao, He-ping Li

**Affiliations:** a Department of Architectural Environment, and Energy Engineering, Henan University of Urban Construction, Pingdingshan 467044, People’s Republic of China; b Department of Chemistry and Chemical Engineering, Henan University of Urban Construction, Pingdingshan 467044, People’s Republic of China; c School of Chemistry and Biological Engineering, Guilin University of Technology, People’s Republic of China

## Abstract

The title Schiff base compound, C_9_H_12_N_2_O_2_, was obtained from a condensation reaction of butan-2-one and furan-2-carbohydrazide. The furan ring and the hydrazide fragment are roughly planar, the largest deviation from the mean plane being 0.069 (2)Å, but the butanyl­idene group is twisted slightly with respect to this plane by a dihedral angle of 5.2 (3)°. In the crystal, inter­molecular N—H⋯O hydrogen bonds link pairs of inversion-related mol­ecules, forming dimers of *R*_2_^2^(8) graph-set motif.

## Related literature

For general properties of Schiff bases, see: Kahwa *et al.* (1986 ▶); Santos *et al.* (2001 ▶). For related structures containing the furan-2-carbohydrazide fragment, see: Jing *et al.* (2007*a* ▶,*b* ▶); Yao & Jing (2007 ▶); Bakir & Gyles (2003 ▶); Tai *et al.* (2007*a* ▶,*b* ▶); Zhou *et al.* (2007 ▶); Butcher *et al.* (2007 ▶); Zhao *et al.* (2007 ▶). For hydrogen-bond motifs, see: Bernstein *et al.* (1995 ▶); Etter *et al.* (1990 ▶).
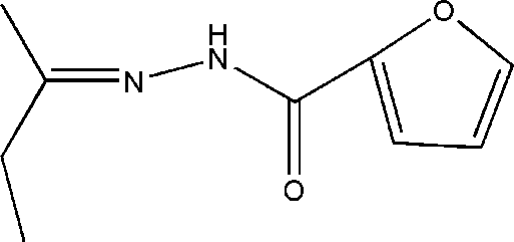


## Experimental

### 

#### Crystal data

C_9_H_12_N_2_O_2_
*M* *_r_* = 180.21
Monoclinic, 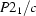

*a* = 8.2664 (15) Å
*b* = 16.6687 (13) Å
*c* = 7.5396 (11) Å
β = 113.171 (19)°
*V* = 955.1 (2) Å^3^
*Z* = 4
Mo *K*α radiation
μ = 0.09 mm^−1^
*T* = 293 K
0.21 × 0.19 × 0.17 mm

#### Data collection

Bruker SMART CCD area-detector diffractometer
Absorption correction: multi-scan (*SADABS*; Bruker, 1998 ▶) *T*_min_ = 0.978, *T*_max_ = 0.982
4182 measured reflections
1955 independent reflections
761 reflections with *I* > 2σ(*I*)
*R*_int_ = 0.040

#### Refinement

*R*[*F* ^2^ > 2σ(*F* ^2^)] = 0.044
*wR*(*F* ^2^) = 0.101
*S* = 0.74
1955 reflections
120 parameters
H-atom parameters constrained
Δρ_max_ = 0.17 e Å^−3^
Δρ_min_ = −0.17 e Å^−3^

### 

Data collection: *SMART* (Bruker, 1998 ▶); cell refinement: *SAINT* (Bruker, 1998 ▶); data reduction: *SAINT*; program(s) used to solve structure: *SHELXTL* (Sheldrick, 2008 ▶); program(s) used to refine structure: *SHELXTL*; molecular graphics: *SHELXTL*; software used to prepare material for publication: *SHELXTL*.

## Supplementary Material

Crystal structure: contains datablocks global, I. DOI: 10.1107/S1600536810038018/dn2605sup1.cif

Structure factors: contains datablocks I. DOI: 10.1107/S1600536810038018/dn2605Isup2.hkl

Additional supplementary materials:  crystallographic information; 3D view; checkCIF report

## Figures and Tables

**Table 1:** Hydrogen-bond geometry (Å, °)

*D*—H⋯*A*	*D*—H	H⋯*A*	*D*⋯*A*	*D*—H⋯*A*
N1—H1⋯O2^i^	0.86	2.16	2.981 (2)	160

Symmetry code: (i) 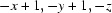.

**Fig. 1.**
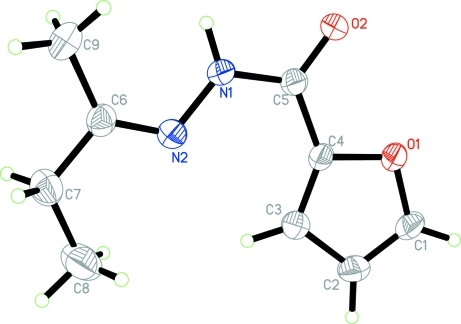
Molecular view of the title compound with the atom labeling scheme. Displacement ellipsoids are drawn at the 30% probability level. H atoms are represented as small sphere of arbitrary radii.

**Fig. 2.**
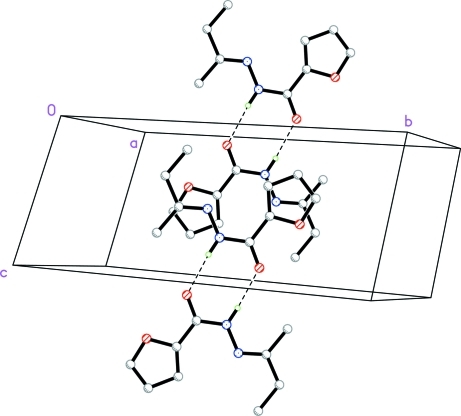
Partial packing view showing the formation of dimer through N-H···O hydogen bonds shown as dashed lines. H atoms not involved in hydrogen bondings have been omitted for clarity.

## supplementary crystallographic information

### Comment

The chemistry of Schiff base has attracted a great deal of interest in recent years. These compounds play an important role in the development of various proteins and enzymes (Kahwa *et al.*, 1986; Santos *et al.*, 2001). In this paper, we synthesized the title compound and reported its crystal structure of the title compound.

The molecular structure of (I) adopts an E conformation with respect to the C=N double bond (Fig.1). The furan ring and the the C5/N1/N2/C6 group are roughly planar with the largest deviation from the mean plane being 0.069 (2)Å but the butan C6/C7/C8/C9 group is slightly twisted with respect to this plane by a dihedral angle of 5.2 (3)°. Distances and bond angles within the furan and the hydrazide moiety agree with related structures found in the literature (Jing *et al.,* 2007*a*,*b*; Yao & Jing, 2007; Bakir & Gyles, 2003; Tai *et al.*, 2007*a*,*b*; Zhou *et al.*, 2007; Butcher *et al.*, 2007; Zhao *et al.*, 2007.

Intermolecular N—H···O hydrogen bonds link the molecules two by two around inversion centers to form dimers with a R_2_^2^(8) graph set motif (Etter *et al.*, 1990; Bernstein *et al.*, 1995) (Table 1, Fig. 2).

### Experimental

Furan-2-carbohydrazine (1 mmol, 0.126 g) was dissolved in anhydrous ethanol (10 ml), The mixture was stirred for several minitutes at 351k, butan-2-one(1 mmol, 0.072 g) in ethanol (8 mm l) was added dropwise and the mixture was stirred at refluxing temperature for 2 h. The product was isolated and recrystallized from methanol/dicholomethane(1:1), colorless single crystals of (I) was obtained after 3 d.

### Refinement

All H atoms attached to C atoms and N atom were fixed geometrically and treated as riding with C—H = 0.96 Å (methyl), 0.97 Å (methylene), 0.93Å (aromatic) and N—H = 0.86 Å with U_iso_(H) = 1.2U_eq_(C or N) or U_iso_(H) = 1.5U_eq_(Cmethyl).

### Crystal data

**Table:**

C_9_H_12_N_2_O_2_	*F*(000) = 384
*M**_r_* = 180.21	*D*_x_ = 1.253 Mg m^−^^3^
Monoclinic, *P*2_1_/*c*	Mo *K*α radiation, λ = 0.71073 Å
Hall symbol: -P 2ybc	Cell parameters from 1520 reflections
*a* = 8.2664 (15) Å	θ = 3.1–28.8°
*b* = 16.6687 (13) Å	µ = 0.09 mm^−^^1^
*c* = 7.5396 (11) Å	*T* = 293 K
β = 113.171 (19)°	Block, colorless
*V* = 955.1 (2) Å^3^	0.21 × 0.19 × 0.17 mm
*Z* = 4	

### Data collection

**Table:**

Bruker SMART CCD area-detector diffractometer	1955 independent reflections
Radiation source: fine-focus sealed tube	761 reflections with *I* > 2σ(*I*)
graphite	*R*_int_ = 0.040
ω scans	θ_max_ = 26.4°, θ_min_ = 3.2°
Absorption correction: multi-scan (*SADABS*; Bruker, 1998)	*h* = −11→9
*T*_min_ = 0.978, *T*_max_ = 0.982	*k* = −18→21
4182 measured reflections	*l* = −6→9

### Refinement

**Table:**

Refinement on *F*^2^	Primary atom site location: structure-invariant direct methods
Least-squares matrix: full	Secondary atom site location: difference Fourier map
*R*[*F*^2^ > 2σ(*F*^2^)] = 0.044	Hydrogen site location: inferred from neighbouring sites
*wR*(*F*^2^) = 0.101	H-atom parameters constrained
*S* = 0.74	*w* = 1/[σ^2^(*F*_o_^2^) + (0.0434*P*)^2^] where *P* = (*F*_o_^2^ + 2*F*_c_^2^)/3
1955 reflections	(Δ/σ)_max_ = 0.003
120 parameters	Δρ_max_ = 0.17 e Å^−^^3^
0 restraints	Δρ_min_ = −0.17 e Å^−^^3^

### Special details

**Table:**

Geometry. All esds (except the esd in the dihedral angle between two l.s. planes) are estimated using the full covariance matrix. The cell esds are taken into account individually in the estimation of esds in distances, angles and torsion angles; correlations between esds in cell parameters are only used when they are defined by crystal symmetry. An approximate (isotropic) treatment of cell esds is used for estimating esds involving l.s. planes.
Refinement. Refinement of *F*^2^ against ALL reflections. The weighted *R*-factor *wR* and goodness of fit *S* are based on *F*^2^, conventional *R*-factors *R* are based on *F*, with *F* set to zero for negative *F*^2^. The threshold expression of *F*^2^ > σ(*F*^2^) is used only for calculating *R*-factors(gt) *etc*. and is not relevant to the choice of reflections for refinement. *R*-factors based on *F*^2^ are statistically about twice as large as those based on *F*, and *R*- factors based on ALL data will be even larger.

### Fractional atomic coordinates and isotropic or equivalent isotropic displacement parameters (Å^2^)

**Table:**

	*x*	*y*	*z*	*U*_iso_*/*U*_eq_	
O1	0.4993 (2)	0.32297 (8)	0.4042 (2)	0.0598 (5)	
O2	0.4694 (2)	0.41298 (8)	0.1056 (2)	0.0670 (6)	
N1	0.6592 (3)	0.50672 (9)	0.2784 (3)	0.0511 (6)	
H1	0.6497	0.5314	0.1746	0.061*	
N2	0.7678 (3)	0.53804 (11)	0.4554 (3)	0.0505 (6)	
C1	0.5264 (4)	0.29006 (14)	0.5768 (4)	0.0586 (8)	
H1B	0.4800	0.2411	0.5932	0.070*	
C2	0.6274 (4)	0.33618 (14)	0.7195 (4)	0.0638 (8)	
H2B	0.6641	0.3262	0.8509	0.077*	
C3	0.6686 (3)	0.40406 (13)	0.6316 (4)	0.0582 (7)	
H3A	0.7383	0.4473	0.6956	0.070*	
C4	0.5899 (3)	0.39476 (11)	0.4416 (3)	0.0437 (6)	
C5	0.5674 (3)	0.43790 (12)	0.2653 (3)	0.0473 (7)	
C6	0.8540 (3)	0.60130 (13)	0.4535 (3)	0.0502 (7)	
C7	0.9668 (4)	0.63698 (13)	0.6436 (4)	0.0669 (8)	
H7A	0.9302	0.6921	0.6457	0.080*	
H7B	1.0872	0.6381	0.6529	0.080*	
C8	0.9648 (4)	0.59519 (17)	0.8197 (4)	0.0933 (10)	
H8A	1.0343	0.6251	0.9329	0.140*	
H8B	1.0128	0.5422	0.8278	0.140*	
H8C	0.8459	0.5916	0.8108	0.140*	
C9	0.8539 (4)	0.64420 (13)	0.2796 (4)	0.0806 (10)	
H9A	0.8447	0.6059	0.1810	0.121*	
H9B	0.9613	0.6740	0.3134	0.121*	
H9C	0.7557	0.6803	0.2325	0.121*	

### Atomic displacement parameters (Å^2^)

**Table:**

	*U*^11^	*U*^22^	*U*^33^	*U*^12^	*U*^13^	*U*^23^
O1	0.0962 (15)	0.0377 (8)	0.0454 (11)	−0.0067 (9)	0.0276 (10)	0.0008 (7)
O2	0.1069 (16)	0.0467 (9)	0.0373 (11)	−0.0157 (9)	0.0174 (11)	−0.0023 (8)
N1	0.0748 (15)	0.0400 (10)	0.0386 (12)	−0.0059 (11)	0.0224 (11)	0.0015 (9)
N2	0.0599 (15)	0.0458 (11)	0.0440 (13)	−0.0015 (10)	0.0186 (11)	−0.0015 (9)
C1	0.085 (2)	0.0456 (13)	0.0495 (18)	−0.0007 (14)	0.0312 (16)	0.0107 (12)
C2	0.077 (2)	0.0659 (16)	0.0435 (17)	−0.0090 (15)	0.0182 (16)	0.0110 (13)
C3	0.067 (2)	0.0543 (14)	0.0472 (17)	−0.0152 (13)	0.0155 (15)	0.0028 (12)
C4	0.0572 (18)	0.0318 (12)	0.0431 (15)	0.0011 (11)	0.0208 (13)	0.0017 (10)
C5	0.0661 (19)	0.0369 (13)	0.0406 (16)	0.0038 (13)	0.0228 (15)	−0.0010 (11)
C6	0.0518 (18)	0.0415 (13)	0.0561 (17)	0.0021 (13)	0.0199 (14)	0.0008 (11)
C7	0.060 (2)	0.0655 (16)	0.069 (2)	−0.0095 (14)	0.0184 (17)	−0.0050 (14)
C8	0.092 (3)	0.123 (2)	0.059 (2)	−0.0321 (19)	0.0227 (18)	−0.0138 (18)
C9	0.101 (3)	0.0641 (17)	0.075 (2)	−0.0200 (16)	0.0328 (19)	0.0117 (14)

### Geometric parameters (Å, °)

**Table:**

O1—C1	1.347 (2)	C4—C5	1.457 (3)
O1—C4	1.381 (2)	C6—C7	1.492 (3)
O2—C5	1.229 (2)	C6—C9	1.493 (3)
N1—C5	1.357 (3)	C7—C8	1.505 (3)
N1—N2	1.384 (2)	C7—H7A	0.9700
N1—H1	0.8600	C7—H7B	0.9700
N2—C6	1.276 (3)	C8—H8A	0.9600
C1—C2	1.319 (3)	C8—H8B	0.9600
C1—H1B	0.9300	C8—H8C	0.9600
C2—C3	1.419 (3)	C9—H9A	0.9600
C2—H2B	0.9300	C9—H9B	0.9600
C3—C4	1.329 (3)	C9—H9C	0.9600
C3—H3A	0.9300		
			
C1—O1—C4	106.56 (17)	N2—C6—C9	126.8 (2)
C5—N1—N2	121.40 (19)	C7—C6—C9	115.9 (2)
C5—N1—H1	119.3	C6—C7—C8	116.3 (2)
N2—N1—H1	119.3	C6—C7—H7A	108.2
C6—N2—N1	117.00 (19)	C8—C7—H7A	108.2
C2—C1—O1	111.2 (2)	C6—C7—H7B	108.2
C2—C1—H1B	124.4	C8—C7—H7B	108.2
O1—C1—H1B	124.4	H7A—C7—H7B	107.4
C1—C2—C3	106.0 (2)	C7—C8—H8A	109.5
C1—C2—H2B	127.0	C7—C8—H8B	109.5
C3—C2—H2B	127.0	H8A—C8—H8B	109.5
C4—C3—C2	107.7 (2)	C7—C8—H8C	109.5
C4—C3—H3A	126.1	H8A—C8—H8C	109.5
C2—C3—H3A	126.1	H8B—C8—H8C	109.5
C3—C4—O1	108.49 (19)	C6—C9—H9A	109.5
C3—C4—C5	139.3 (2)	C6—C9—H9B	109.5
O1—C4—C5	112.16 (19)	H9A—C9—H9B	109.5
O2—C5—N1	119.4 (2)	C6—C9—H9C	109.5
O2—C5—C4	121.6 (2)	H9A—C9—H9C	109.5
N1—C5—C4	118.9 (2)	H9B—C9—H9C	109.5
N2—C6—C7	117.3 (2)		

### Hydrogen-bond geometry (Å, °)

**Table:**

*D*—H···*A*	*D*—H	H···*A*	*D*···*A*	*D*—H···*A*
N1—H1···O2^i^	0.86	2.16	2.981 (2)	160

Symmetry codes: (i) −*x*+1, −*y*+1, −*z*.
